# 

**Published:** 1912-11-23

**Authors:** 


### Two Bacterial Researches.

Two common and important diseases are the subject of brief communications to the *Lancet* on their bacterial aspects. Dr. G. C. Purvis, of Cape Colony, finds that sodium salicylate affords a useful method of distinguishing between Bacillus typhosus and B. coli, because 1 per cent. of it inhibits the growth of the former completely in culture media, whereas it has no such effect upon the latter. He further suggests that possibly this fact may be turned to account in the treatment of typhoid fever; and that it would be worth while to give patients small doses of the salicylate in capsules which would dissolve only in the small intestine.

The other research is one by Drs. Poynton and Paine into the ætiology of appendicitis. These authors last year published an account of experimental appendicitis produced in animals by injection into the blood stream of the Diplococcus rheumaticus first described at the beginning of the century by these authors. They have now had the opportunity of observing a patient who was suffering from acute appendicitis and also from tonsillitis. From the throat they were able to isolate a streptodiplococcus; and from the appendix, removed at operation and placed in a sterile tube, they also isolated an organism apparently exactly similar. With the culture from the throat they produced acute arthritis in animals by intravenous injection; these investigations are being continued, but apparently this organism has not produced appendicitis so far. With the similar organism from the appendix they produced arthritis in five rabbits out of six, and appendicitis in one. A further important observation was that the (human) appendix showed diplococci in the mucous and submucous coats; and also, more numerous still, in the serous membrane. The authors conclude that "a cause of appendicitis may be a streptoccocal invasion through the blood stream from a follicular tonsillitis." This conclusion would have been strengthened if the authors had demonstrated that the patient's blood contained the streptococcus, and if the organism from the throat had been shown to produce appendicitis experimentally.

### Books Received.

W. B. CLIVE: "Science French Course," by C. W. Paget Moffatt.

WILLIAMS AND NORGATE: "The Human Body," by Professor A. Keith, M.D.

J. HEYWOOD, LTD. (Manchester): "Electricity for Everybody," by G. R. Peers.

OXFORD MEDICAL PUBLICATIONS: "Surgery of the Skull and Brain," by L. Bathe Rawling.

CORNISH BROS.: "Coprostasis: Its Cause, Prevention, and Treatment," by Sir James Sawyer.

J. AND A. CHURCHILL: "Mother and Baby," by Selina F. Fox, M.D., B.S.; "On Alcoholism: its Clinical Aspects and Treatment," by Francis Hare, M.D.

### Buildings Contemplated and in Progress.

Barking.—The Local Government Board is inquiring into an application for sanction to a loan of £2,275 in connection with Barking Urban District Council Infectious Hospital.

Devonport.—The Local Government Board is inquiring into a proposed loan of £4,500 for the extension of the Town Council's Infectious Hospital grounds.

Forden.—The Montgomeryshire County Council has resolved to purchase the workhouse premises and land at Forden at a cost of £6,000, for conversion into a county lunatic asylum.

Huddersfield.—A new pavilion block is projected at Huddersfield for the Colne and Holme Joint Hospital Committee.

Kettering.—The Joint Hospital Board propose to borrow £1,400 in connection with the infectious diseases hospital. The Local Government Board has fixed the date for an inquiry.

Long Bay.—The Council of the British Medical Association has suggested the extension of Long Bay Hospital and the erection of a new hospital at North Sydney.

Marske.—The Guisborough Rural District Council has approved plans for an isolation hospital near Marske.

Neath.—The Neath Guardians propose to borrow a further £4,000 for laundry and engineering offices at the new graded infirmary.

Straits Settlements.—The 1913 Budget of the Straits Settlements provides for additions and alterations to the general hospital, including electric lighting, 135,000 dols.; installation of water-carriage sewerage system at hospitals, gaols, and asylums, 90,000 dols.; and 100,000 dols. towards the cost of a new lunatic asylum, estimated to cost ultimately 750,000 dols. A new hospital at Jasin, Malacca, is estimated to cost 70,000 dols.

Watrons.—The Grand Trunk Pacific Railway has arranged for the establishment of an hotel and sanatorium at Lake Manitou, at an estimated cost of £50,000.

Windsor.—The Secretary of State for War invites tenders for a new hospital, together with contingent works, at Combermere Barracks, Windsor. Particulars may be had from the Director of Barrack Construction, London.

Winwick (Warrington).—Tenders are under consideration for an extension of the nurses' home at the Lancashire County Mental Hospital, Winwick. An application for sanction to a loan of £2,200 is being made.

Yorkshire.—The West Riding County Council has decided to erect a 150-bed sanatorium and 50 shelters at a cost of £26,000, towards which a Government grant of £16,300 is anticipated.

### A COUNTY COUNCIL'S GIFT TO A MEDICAL STUDENT.

It is interesting to note that in order to supplement a scholarship of £70 won in May last at Westminster Hospital, the Berkshire County Council have granted a special Scholarship of £50 to Mr. Frank Portas, who some four years ago left the elementary school at Windsor. This grant will thus materially assist him in his medical studies.

### BUREAU OF INFORMATION.

### Rules for Correspondents.

1. Every letter, on whatever subject, **must be accompanied by the coupon** to be cut from the back cover (inside page) of The Hospital, current issue, and must contain the name and address of the correspondent with pseudonym for publication if desired.

2. Replies by post cannot be given, save under exceptional circumstances at the Editor's discretion.

3. Letters from Approved Homes in reply to special needs published in the Bureau should state terms and full particulars, and be sent prepaid under cover to the Editor of the Bureau with coupon and name enclosed for identification.

4. Proprietors of Homes which have not yet been entered on the List of Approved Homes, but have spare accommodation likely to suit special needs, are invited to write for an application form for registration. The fee for registration, which includes two announcements of the Home in the Bureau, is 5s.

5. Special needs of every description relating to those who are sick or in difficulties are made known in these columns **free of charge.**

6. All communications to be addressed to The Editor of The Hospital, 28 Southampton Street, Strand, London, W.C., and marked "Bureau of Information."

### Needs and Helpers.

**Open-Air Treatment for Girl.**—Greatly obliged for your kind and helpful suggestion which we hope may be of use also to another reader. We have sent Sister S. W. your address, and no doubt she will communicate with you direct. The accommodation your Home offers at moderate terms for early phthisical cases is greatly in request.—Spes.

**Knitting Machine Wanted.**—Has any reader a good knitting machine to dispose of, second-hand, for the use of a lady in delicate health who is an expert knitter and desires to contribute to her own support through this means?

### Training and Employment.

**Midwifery under Doctor.**—We are afraid you may find it difficult to obtain continuous employment as a maternity nurse under the circumstances. Medical men prefer maternity nurses who have also had full general training. Aim at very moderate fees at first and take whatever is offered with a view to getting known. Let the doctors in the neighbourhood know that you are prepared to meet patients in poor circumstances as regards your fees, and you will soon get cases. But you cannot attempt to compete with the nurses who have higher training on the same terms.—Nurse W.

### Homes Wanted.

**For Elderly Person.**—Home wanted in homely surroundings by lady who has very small means and does not wish to live in rooms or alone. Quite simple accommodation, sharing meals, etc., with a friendly nurse. State lowest terms to E. N. (134a).

**For Invalid Superintendent.**—The patient has broken down in health while carrying on small nursing home, and, though now recovering, the brain is slightly affected as a consequence of fits at the onset of the illness—*not* true epilepsy. She is bright and capable of giving some assistance in serving, etc. Her age is a little over forty. Home within easy reach of London preferred; very moderate terms. Replies to K. M. L. (135a).

### Nursing in the Empire and Abroad.

**Conditions in Bloemfontein.**—Private nurses undoubtedly command very high fees in this spot, five guineas and more per week, with all found. There is a distinct demand among the doctors out there for first-class nurses trained in modern abdominal and aseptic surgery, and good midwifery and fever nurses are also in request. There is a leaning towards nurses trained in well-known hospitals. If you really think of going out we can supply the name of an authority on the spot from whom you can obtain full information. There are a good many nurses already working here and there would be competition.—R. U. K.

**The East Africa Nursing Association.**—The head quarters of this Association are at Nairobi, *via* Mombasa, East Africa, where there is a Nursing Home. Fees are from three to five guineas a week, and there is a fairly steady demand for nurses. Communicate with the Secretary as above.—Lily L.

**Nursing Home at Malta.**—The newly founded Zannit Clapp Hospital here meets a great need, and constitutes a real protection to those like yourself who purpose making a long stay in Malta. It is erected on high ground and contains comfortable airy rooms for private patients as well as some well-designed wards. There is a Home for trained nurses in connection with the hospital, and you cannot do better than apply here should any occasion arise for their services during the winter. The address is Nursing Sisters, L. C. M., Casa Leone XIII., Malta.—Artillery.

**Nursing in Greece.**—If you want to see Athens choose some other moment. We are advised that there is rather an anti-English feeling just now prevailing there, and that, consequently, Greeks would be ill disposed to employ Englishwomen. Besides, the country is thoroughly unsettled, and it is doubtful if you could get a passage over.—Earnest.

### Approved Homes.

Note.—*No Home is added to the List without direct medical and other testimony to its good management.*

**Clapham Common, S.W.**—6 Crescent Grove. Principal: Miss Starey. Home for ladies—medical, surgical, and maternity, but principally for the aged. Established nineteen years. Large grounds and country cottage dépendance at Amersham-on-Hill, Bucks. Very moderate terms. Telephone: 429 Battersea.

**Surrey.**—Gothic House, Tadworth. Principal: Miss Haines, certified nurse masseuse. Small, comfortable rest-house, with private sitting-rooms; 600 feet above sea-level, on extensive common near Walton Heath Golf Links and Epsom Downs; within an hour of London. Inclusive terms or rooms.

**Bedfordshire.**—Burnham House, Toddington, near Dunstable. Principals: Mr. and Mrs. A. V. Drewitt Pinchin. Private Home for four mental or epileptic patients (male). Special experience and skill with difficult youths. Healthy, bracing situation, with good garden.

### Letter-Box.

L. J. L., Brockley.—We are unable to introduce to patients any but Homes on our List.

Mrs. B., Southwell.—The Nursing Home in question is one of the finest in the country, and its administration leaves nothing to be desired.

Consul-General, Ispahan; Consul, Rosario; Medical Director, Salisbury, Rhodesia; Acting British Vice-Consul, Yokohama; Principal Medical Officer, Kuching; Principal Medical Officer, Freetown, Sierra Leone; E. W. Mills, Canton.—We are greatly obliged for the valuable and detailed information you have kindly furnished.

Snarkey.—We note that no further replies are needed.

J. M. P.—We have handed your letter to Sister Rosa Smith, 15 Buckingham Street, Strand.

**Communications have been received and attended to from:—**

Rev. W. J. W., Pentir, Bangor; Rev. J. L. D., Menai Bridge; Miss E. Ll., Handsworth; Mrs. R., Leytonstone;

Rev. H. E. Y. B., Alston; Miss W., Seaton; Mr. R. E., Bowes Park; Miss S., Blyth; Miss A. E. D., Highbury; Miss N., Bath; Rev. C. H. L., Suffolk; Miss L. J. C., Sutton; Nurse Foreman; Librarian, Oldham; Mrs. G., Blackpool; Nurse Carter; Nurse D., Cheltenham; Mr. D. P., Beds.

**The Editor begs to acknowledge receipt of communications, with enclosures, from:—**

Mrs. W.; Miss M. L., Worthing; Miss K. H., Clacton.

**The Editor is much indebted for testimonials received, in response to, inquiries from:—**

Miss L. S.; Dr. S., Clapham Common; Dr. McA.; Mr. P. P., Ipswich; Miss D. G., Highgate; Dr. B. H., Hornsey; Matron E. B.; Dr. W., Sherborne; Dr. S., Walsall; Mrs. W., Brondesbury.

**Letters forwarded as requested from:—**

Miss L. A. H., Mrs. R., Miss R., Mr. M. E. M., Nurse Watson, Nurse R. M., Miss L. S., and others.

**A leaflet containing particulars of the Bureau will be forwarded free on application.**

### Gifts and Bequests.

Queen Alexandra has sent £20 to the Rebuilding Fund of the Chelsea Hospital for Women.

The Princess Victoria has sent a donation of £5 to the Rebuilding Fund of the Chelsea Hospital for Women.

Mr. James Ferguson Pullar, younger brother of the late Sir Robert Pullar, dyer, has left £500 to the Perth County and City Royal Infirmary.

Mr. Herbert Sayer Tew, a banker, has bequeathed £10,000 to the Clayton Hospital, Wakefield, and £5,000 to the Pontefract General Dispensary.

### A PHYSICIAN'S BEQUESTS TO THE SEAMEN'S HOSPITAL.

Dr. Andrew Duncan, whose death was announced in these columns on October 26, has made the following bequests to the Seamen's Hospital Society:—£100 to provide a prize to be awarded to the nurse at the Albert Dock Hospital who shall pass out first at the annual examination in tropical medical nursing; £150 to provide a medal to be awarded annually to a student in the school in such manner as the staff think best; and all his books on tropical subjects and climate. It is interesting to note that Dr. Duncan served as house physician at the "Dreadnought" Hospital, Greenwich, in 1874.

